# Chronic Hypoxia during Gestation Enhances Uterine Arterial Myogenic Tone via Heightened Oxidative Stress

**DOI:** 10.1371/journal.pone.0073731

**Published:** 2013-09-16

**Authors:** Daliao Xiao, Xiang-Qun Hu, Xiaohui Huang, Jianjun Zhou, Sean M. Wilson, Shumei Yang, Lubo Zhang

**Affiliations:** 1 Center for Perinatal Biology, Division of Pharmacology, Department of Basic Sciences, Loma Linda University School of Medicine, Loma Linda, California, United States of America; 2 Department of Obstetrics and Gynecology, Nanjing Drum Tower Hospital, Nanjing University Medical School, Nanjing, China; 3 Department of Chemistry and Biochemistry, California State University, San Bernardino, California, United States of America; University of Kentucky, United States of America

## Abstract

Chronic hypoxia during gestation has profound adverse effects on the adaptation of uteroplacental circulation in pregnancy. Yet, the underlying mechanisms are not fully understood. The present study tested the hypothesis that enhanced production of reactive oxygen species (ROS) in uterine arteries plays a critical role in the maladaptation of uterine circulation associated with chronic hypoxia. Uterine arteries were isolated from nonpregnant and near-term pregnant sheep maintained at sea level (∼300 m) or exposed to high-altitude (3801 m) hypoxia for 110 days. Hypoxia significantly increased ROS production in uterine arteries of pregnant, but not nonpregnant, sheep. This was associated with a significant increase in NADPH oxidase (Nox) 2, but not Nox1 or Nox4, protein abundance and total Nox activity in uterine arteries of pregnant animals. Chronic hypoxia significantly increased pressure-dependent uterine arterial myogenic tone in pregnant sheep, which was abrogated by a Nox inhibitor apocynin. Additionally, the hypoxia-induced increase in myogenic reactivity of uterine arteries to phorbol 12,13-dibutyrate in pregnant sheep was blocked by apocynin and tempol. In consistence with the myogenic responses, the hypoxia-mediated down-regulation of BK_Ca_ channel activity in uterine arteries of pregnant animals was reversed by apocynin. The findings suggest that heightened oxidative stress in uterine arteries plays a key role in suppressing the BK_Ca_ channel activity, resulting in increased myogenic reactivity and maladaptation of uteroplacental circulation caused by chronic hypoxia during gestation.

## Introduction

Uterine vascular myogenic reactivity is a key physiological mechanism in regulating basal vascular tone and uterine blood flow, and reduction in pressure-dependent uterine vascular myogenic tone contributes significantly to the adaptation of uteroplacental circulation in pregnancy [1]–[7]. Increased Ca^2+^-activated K^+^ (BK_Ca_) channel activity plays a key role in attenuating myogenic tone of the uterine artery in pregnancy [8]–[10]. Chronic hypoxia during pregnancy is a common stress to maternal cardiovascular homeostasis and has profound adverse effects on uteroplacental circulation, leading to a 2–4 fold increase in the incidence of preeclampsia and fetal intrauterine growth restriction [11]–[13]. Our recent studies have demonstrated that chronic hypoxia during gestation results in inhibition of BK_Ca_ channel activity and an increase in pressure-dependent myogenic tone and protein kinase C (PKC)-mediated myogenic reactivity of uterine arteries in pregnant sheep [14]–[17]. However, the molecular mechanisms underlying gestational hypoxia-mediated alterations of uterine vascular function are not fully understood.

Reactive oxygen species (ROS), serving as important signaling molecules in vascular smooth muscle cells, mediate numerous physiological processes. Of importance, ROS have been implicated in the pathogenesis of a number of vascular dysfunctions, including pulmonary hypertension and preeclampsia [18]–[20]. Hoffmann et al [21] demonstrated an important causative role for increased ROS in the development of hypertension and in the pathogenesis of preeclampsia in an animal model that spontaneously develops the disease. Although intracellular ROS may be generated by various sources, NADPH oxidase (Nox) appears to be the major ROS generator in the vasculature. Recent studies indicate that hypoxia increases ROS generation *via* Nox signaling pathways, and Nox inhibition reduces hypoxia-mediated responses in the vasculature [22], [23]. In addition, pharmacological inhibition and genetic ablation of ROS generation blocked hypoxia-induced activation of PKC and myogenic response [24], [25]. The role of ROS in the regulation of uterine vascular function in response to chronic hypoxia in gestation has not been investigated, however. Herein, we present evidence that heightened Nox-mediated ROS production suppresses BK_Ca_ channel activity and results in an increase in PKC-mediated myogenic reactivity and myogenic tone of uterine arteries in pregnant sheep acclimatized to long-term high altitude hypoxia.

## Materials and Methods

### Tissue preparation

Uterine arteries were obtained from nonpregnant and near-term (∼140 days' gestation) pregnant sheep maintained at sea level (∼300 m) or exposed to high-altitude (3801 m) hypoxia (arterial Po_2_: 60 mmHg) for 110 days [15]. Animals were anesthetized with thiamylal (10 mg/kg, i.v.) followed by inhalation of 1.5% to 2.0% halothane. An incision was made in the abdomen and the uterus exposed. Uterine arteries were isolated and removed without stretching and placed into a modified Krebs solution. All procedures and protocols were approved by the Institutional Animal Care and Use Committee of Loma Linda University (IACUC#8110004) and followed the guidelines by the National Institutes of Health Guide for the Care and Use of Laboratory Animals.

### Measurement of vascular ROS production

Dihydroethidium (DHE) fluorescence was used to image ROS *in situ*, as described previously [26]–[28]. Briefly, unfixed frozen uterine artery segments were cut into 20-μm thick sections using a Leica CM 3050S cryostat at −20°C. Tissue slides were incubated with DHE (5 µM) at 37°C for 30 min. The slides were viewed with an Olympus BH-2 microscope, and images were captured with a SPOT digital camera imaging system. Total ROS in uterine artery segments were measured with the Oxiselect™ in vitro ROS/RNS assay kit (Cell Biolabs, Inc. San Diego, CA) following the manufacture's instruction, as described previously [27], [28].

### Measurement of myogenic tone

Pressure-dependent myogenic tone of resistance-sized uterine arteries (∼150 µm in diameter) was measured as previously described [14], [15], [29]. Briefly, uterine arteries were dissected, mounted and pressurized in an organ chamber (Living Systems, Burlington, VT). The intraluminal pressure was controlled by a servo-system and arterial diameter was recorded using the SoftEdge Acquisition Subsystem (IonOptix LLC, Milton MA). Following the equilibration period, the intraluminal pressure was increased in a stepwise-manner from 10 to 100 mmHg in 10-mmHg increments. To determine the maximum passive diameter, the passive pressure-diameter relation was conducted in Ca^2+^-free physiologic saline solution containing 3.0 mmol/L of EGTA. The following formula was used to calculate percent myogenic tone at each pressure step: % myogenic tone  =  (D1 – D2)/D1×100, where D1 is the passive diameter in Ca^2+^-free physiologic saline solution (0 Ca^2+^ with 3.0 mmol/L of EGTA) and D2 is the active diameter with normal physiologic saline solution in the presence of extracellular Ca^2+^.

### Contraction studies

Fourth branches of main uterine arteries from both pregnant and nonpregnant sheep were isolated, and cut into 2-mm ring segments and mounted in 10-mL tissue baths containing modified Krebs solution equilibrated with a mixture of 95% O_2_ and 5% CO_2_. Phorbol 12,13-dibutyrate (PDBu)-induced concentration-dependent contractions were obtained by cumulative additions of PDBu in approximate one-half log increments, in the absence or presence of a Nox inhibitor apocynin or a SOD mimetic tempol, as described previous [15], [26], [29].

### Western immunoblotting

Uterine arteries were homogenized in a lysis buffer, followed by centrifugation at 10,000 g at 4°C for 10 min. Supernatants were collected, and samples with equal protein were loaded and separated by SDS-PAGE. Membranes were incubated with rabbit anti-Nox1 (Sigma-Aldrich), mouse anti-Nox2 (BD Biosciences), rabbit anti-Nox4 (Abcam Inc) or anti-HIF-1α (BD Biosciences) antibodies, respectively, followed by a secondary horseradish peroxidase-conjugated antibody [26], [27]. Proteins were visualized with enhanced chemiluminescence reagents, and blots were exposed to Hyperfilm. Results were quantified with the Kodak electrophoresis documentation and analysis system. The target protein abundance was normalized to β-actin.

### Measurement of vascular Nox activity

Nox activity was measured by a lucigenin-derived chemiluminescence assay as described [30]. Briefly, uterine artery was washed with ice-cold PBS and homogenized in cold lysis buffer (20 mmol/L KH2PO4, pH 7.0, 1 mmol/L EGTA, 10 μg/mL aprotinin, 0.5 μg/mL leupeptin, 0.7 μg/mL pepstatin, and 0.5 mmol/L PMSF). The homogenate was centrifuged at 1000 g for 10 minutes at 4°C. The pellet was suspended in a lysis buffer containing protease inhibitors and manually homogenized on ice. Nox activity was measured by a luminescence assay in a 50 mmol/L phosphate buffer, pH 7.0, containing 1 mmol/L EGTA, 150 mmol/L sucrose, 5 μmol/L dark-adapted lucigenin as the electron acceptor, and 100 μmol/L NADPH as the substrate. The chemiluminescent signal was measured using a Synergy HT luminmeter (Biotek, Vermont). The data were normalized to the protein content in each sample.

### Electrophysiology

Smooth muscle cells were enzymatically dissociated from resistance-sized uterine arteries, and whole-cell K^+^ currents were recorded using an EPC 10 patch-clamp amplifier with Patchmaster software (HEKA, Lambrecht/Pfalz, Germany) at room temperature, as previously described [8], [14]. Briefly, cell suspension drops were placed in a recording chamber and adherent cells were continuously superfused with HEPES-buffered physiologic salt solution. Micropipettes were pulled from borosilicate glass and had resistances of 2 to 5 MΩ when filled with the pipette solution containing (mmol/L) 140.0 KCl, 1.0 MgCl_2_, 5.0 Na_2_ATP, 5.0 EGTA, 10.0 HEPES (pH 7.2). Cells were held at –50 mV and whole-cell K^+^ currents were evoked by voltage steps from −60 mV to +80 mV by 10-mV stepwise depolarizing pulses. BK_Ca_ currents were recorded with continuous perfusion of HEPES-buffered physiologic salt solution containing 5 mmol/L 4-aminopyridine (4-AP) that block voltage-gated K^+^ channels. The K^+^ currents were normalized to cell capacitance and were expressed as picoampere per picofarad (pA/pF).

### Data analysis

Results were expressed as means ± SEM obtained from the number (n) of experimental animals given. Concentration-response curves were analyzed by computer-assisted nonlinear regression to fit the data using GraphPad Prism (GraphPad Software, San Diego, CA) to obtain pD_2_ (−log EC_50_) and the maximum response (E_max_). Differences were evaluated for statistical significance (P<0.05) by ANOVA or *t*-test, where appropriate.

## Results

### Chronic hypoxia increased HIF-1α expression

**Figure 1:**
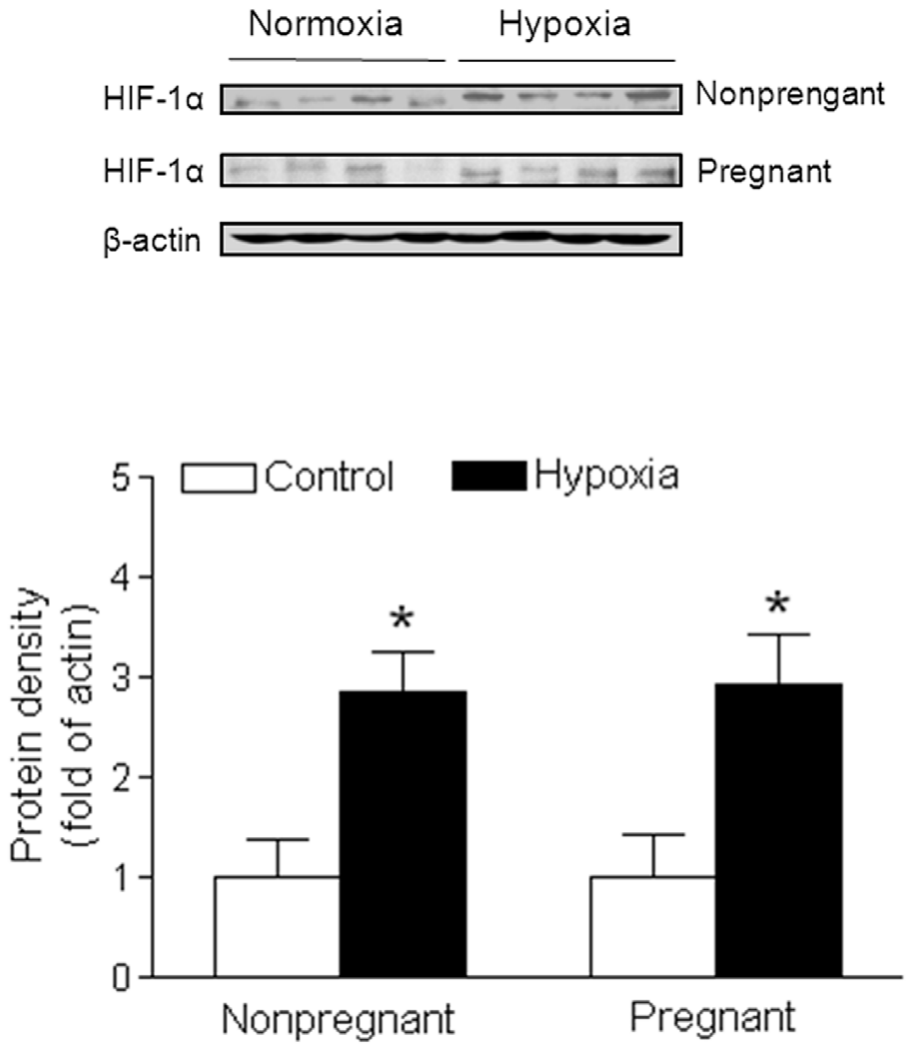
Effect of chronic hypoxia on HIF-1α protein expression in uterine arteries. Uterine arteries were isolated from near-term pregnant and nonpregnant sheep maintained at sea level (control) or exposed to high-altitude hypoxia for 110 days. Protein abundance of HIF-1α was determined by Western blot analyses. Data are means ± SEM of tissues from 4 animals of each group, *, *P*<0.05, versus control.

Hypoxia-inducible factor-1 (HIF-1) is a key transcription factor for response to low oxygen. In order to determine whether chronic hypoxia had direct effect on uterine vascular function, we measured HIF-1α protein expression in uterine artery. As shown in Figure 1, chronic hypoxia significantly enhanced HIF-1α protein levels in both pregnant and nonpregnant uterine arteries as compared with the control ones.

### Chronic hypoxia increased ROS production

**Figure 2:**
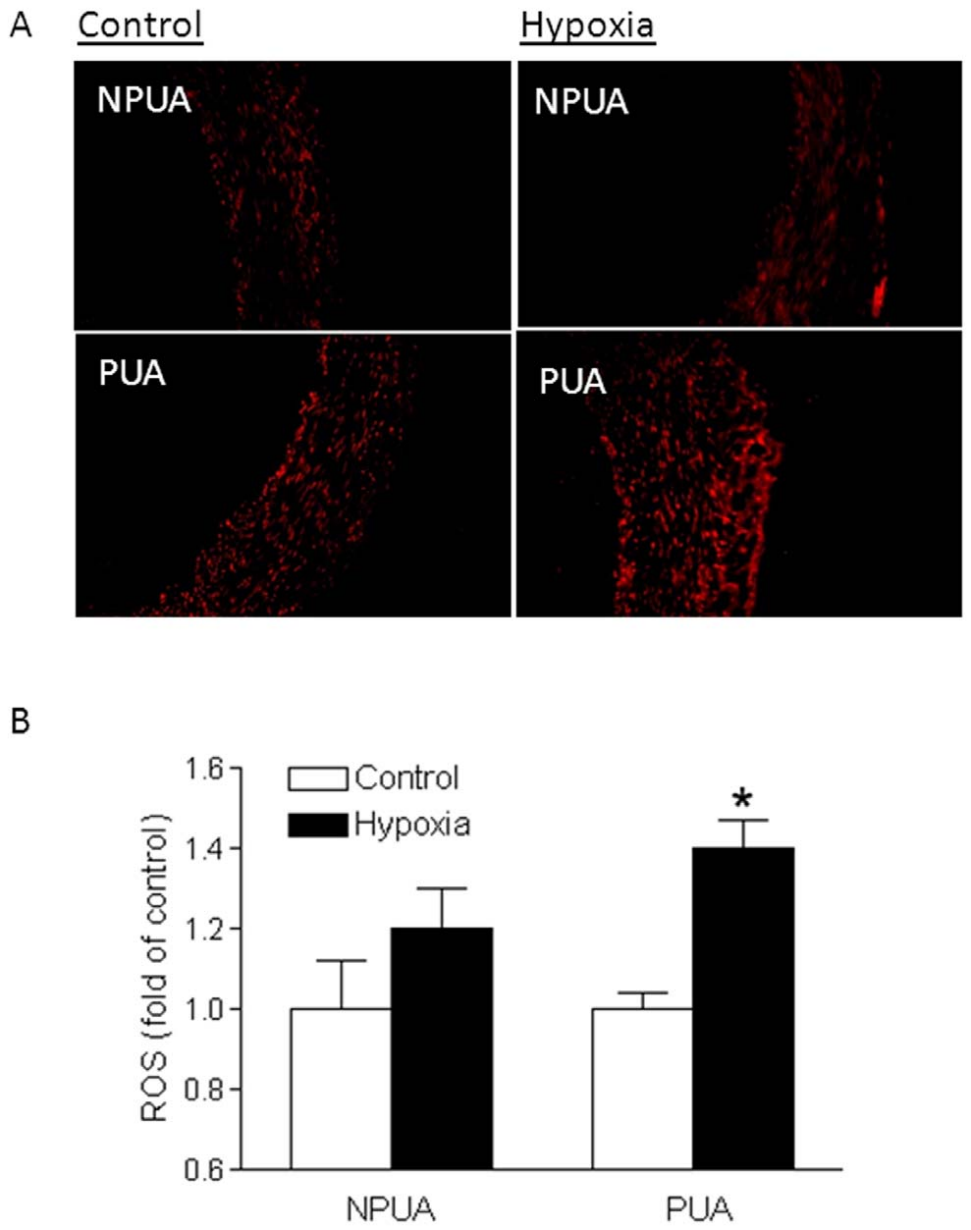
Effect of chronic hypoxia on ROS levels in uterine arteries. Uterine arteries were isolated from nonpregnant (NPUA) and near-term pregnant (PUA) sheep maintained at sea level (control) or exposed to high-altitude hypoxia for 110 days. **A.** ROS detected *in situ* in uterine arterial walls with dihydroethidium fluorescence. **B.** ROS levels measured with a DCF-based quantitative assay kit. Data are means ± SEM of tissues from 5–7 animals of each group. *, *P*<0.05, versus control.

ROS production in uterine arteries was measured *in situ* with superoxide-mediated DHE fluorescence, as well as a fluorescent 2′,7′-dichlorodihydrofluorescein (DCF)-based quantitative assay kit. As shown in Figure 2A, chronic hypoxia increased *in situ* DHE fluorescence in the vascular wall of uterine arteries from pregnant sheep, but not in nonpregnant animals. Consistently, the measurement of ROS with the quantitative assay demonstrated a significant increase in ROS production in uterine arteries of pregnant, but not nonpregnant, animals acclimatized to long-term high altitude hypoxia (Figure 2B).

### Chronic hypoxia upregulated Nox2 expression and total Nox activity

**Figure 3:**
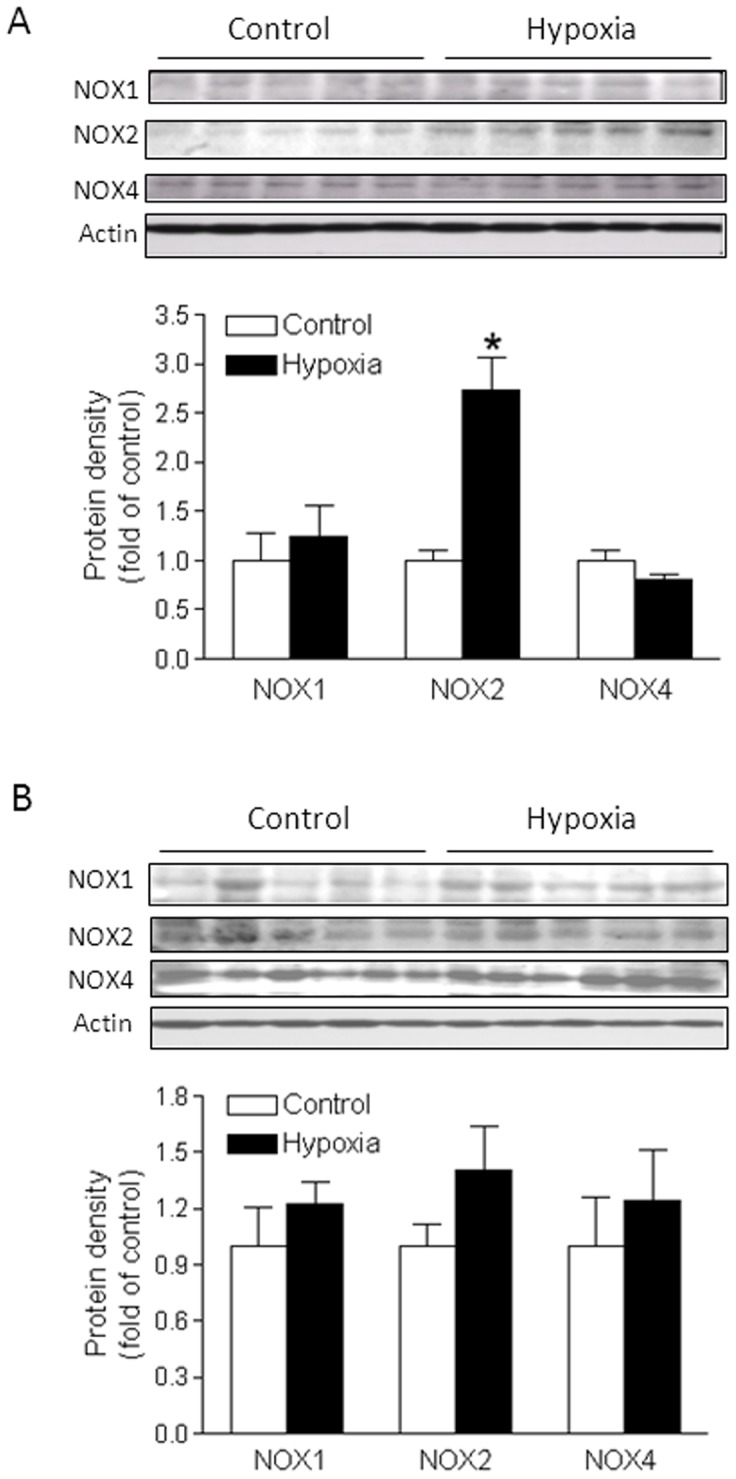
Effect of chronic hypoxia on Nox expression in uterine arteries. Uterine arteries were isolated from near-term pregnant (**A**) and nonpregnant (**B**) sheep maintained at sea level (control) or exposed to high-altitude hypoxia for 110 days. Protein abundance of Nox was determined by Western blot analyses. Data are means ± SEM of tissues from 5 animals of each group, *, *P*<0.05, versus control.

**Figure 4:**
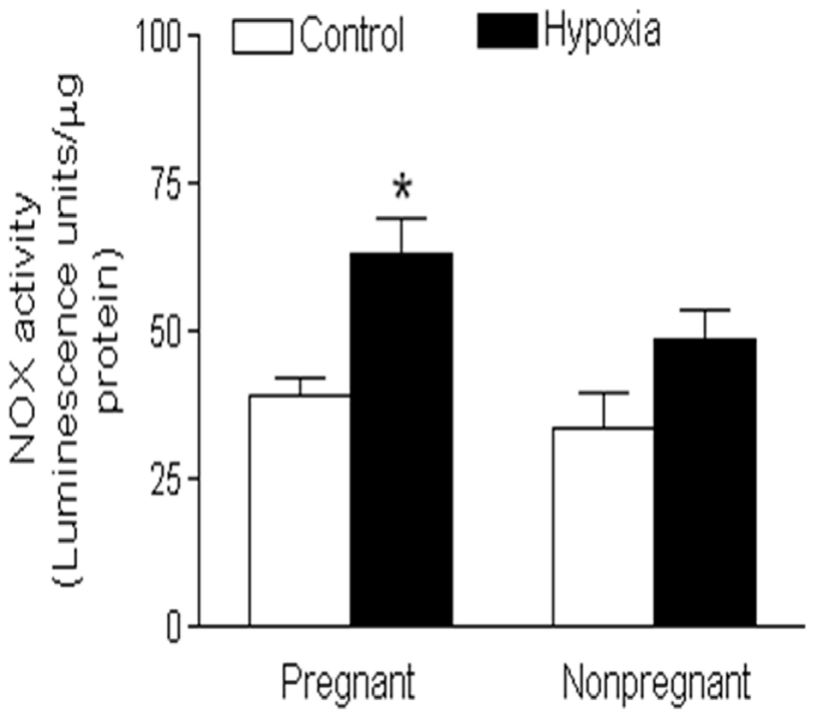
Effect of chronic hypoxia on Nox activity in uterine arteries. Uterine arteries were isolated from near-term pregnant and nonpregnant sheep maintained at sea level (control) or exposed to high-altitude hypoxia for 110 days. NADPH oxidase activity (Nox) was measured by lucigenin luminescence in the uterine arteries. Data are means ± SEM of tissues from 5 animals of each group, *, *P*<0.05, versus control.

Nox is a major source of ROS generation in the vasculature. Among 7 isoforms of the Nox family, Nox1, Nox2, and Nox4 predominantly expressed in the vasculature, and play an important role in the regulation of vascular function. To determine whether chronic hypoxia affects the Nox expression in uterine arteries, protein abundance of Nox1, Nox2, and Nox4 was determined in uterine arteries of both normoxic and hypoxic animals using Western blotting analysis. As shown in Figure 3A, chronic hypoxia had no significant effect on Nox1 and Nox4 protein expression, but significantly increased Nox2 protein abundance in uterine arteries of pregnant sheep. In contrast, none of the three Nox isoforms were significantly altered by chronic hypoxia in uterine arteries of nonpregnant animals (Figure 3B). In addition, chronic hypoxia significantly enhanced total Nox activity in pregnant but not nonpregnant uterine arteries as compared with the control groups (Figure 4).

### Inhibition of ROS abrogated hypoxia-upregulated myogenic tone

**Figure 5:**
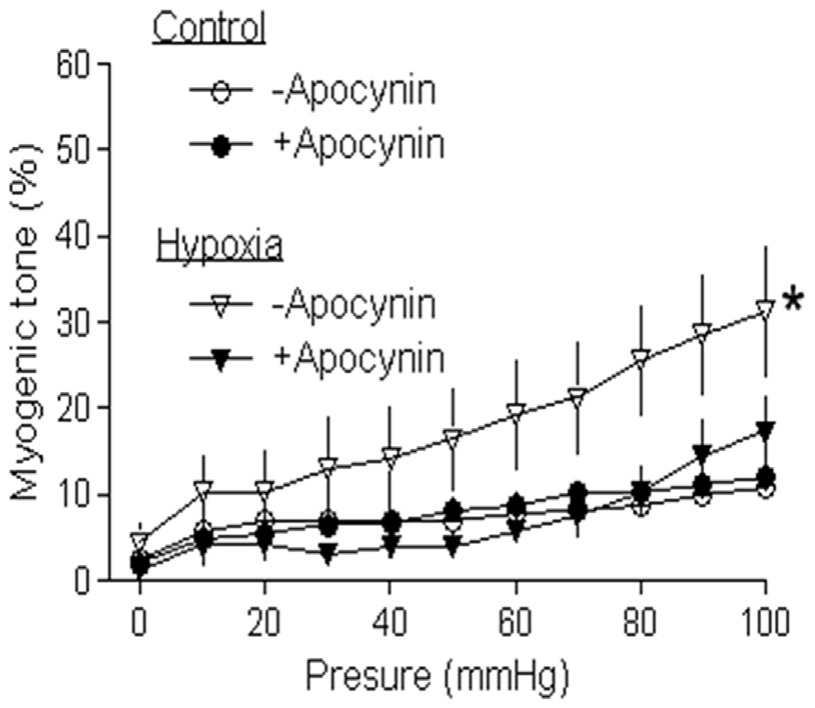
Effect of apocynin on hypoxia-upregulated myogenic tone of uterine arteries. Uterine arteries were isolated from near-term pregnant sheep maintained at sea level (control) or exposed to high-altitude hypoxia for 110 days. Pressure-dependent myogenic tone of uterine arteries was determined in the absence or presence of apocynin (100 µmol/L). Data are means ± SEM of tissues from 5 animals of each group. *, *P*<0.05, versus +apocynin.

Our previous studies demonstrated that pregnancy downregulated pressure-dependent myogenic tone of uterine arteries, which was inhibited by chronic hypoxia during gestation [15], [29]. As shown in Figure 5, in the absence of a Nox inhibitor apocynin, pressure-dependent myogenic tone of uterine arteries in pregnant sheep was significantly upregulated in animals acclimatized to long-term high altitude hypoxia, as compared with the control. Apocynin produced a significant inhibition of pressure-dependent myogenic tone in hypoxic animals. In the presence of apocynin, there was no significant difference in uterine arterial myogenic tone between the control and hypoxic animals.

### Inhibition of ROS abolished hypoxia-enhanced PKC-mediated contractions

**Figure 6:**
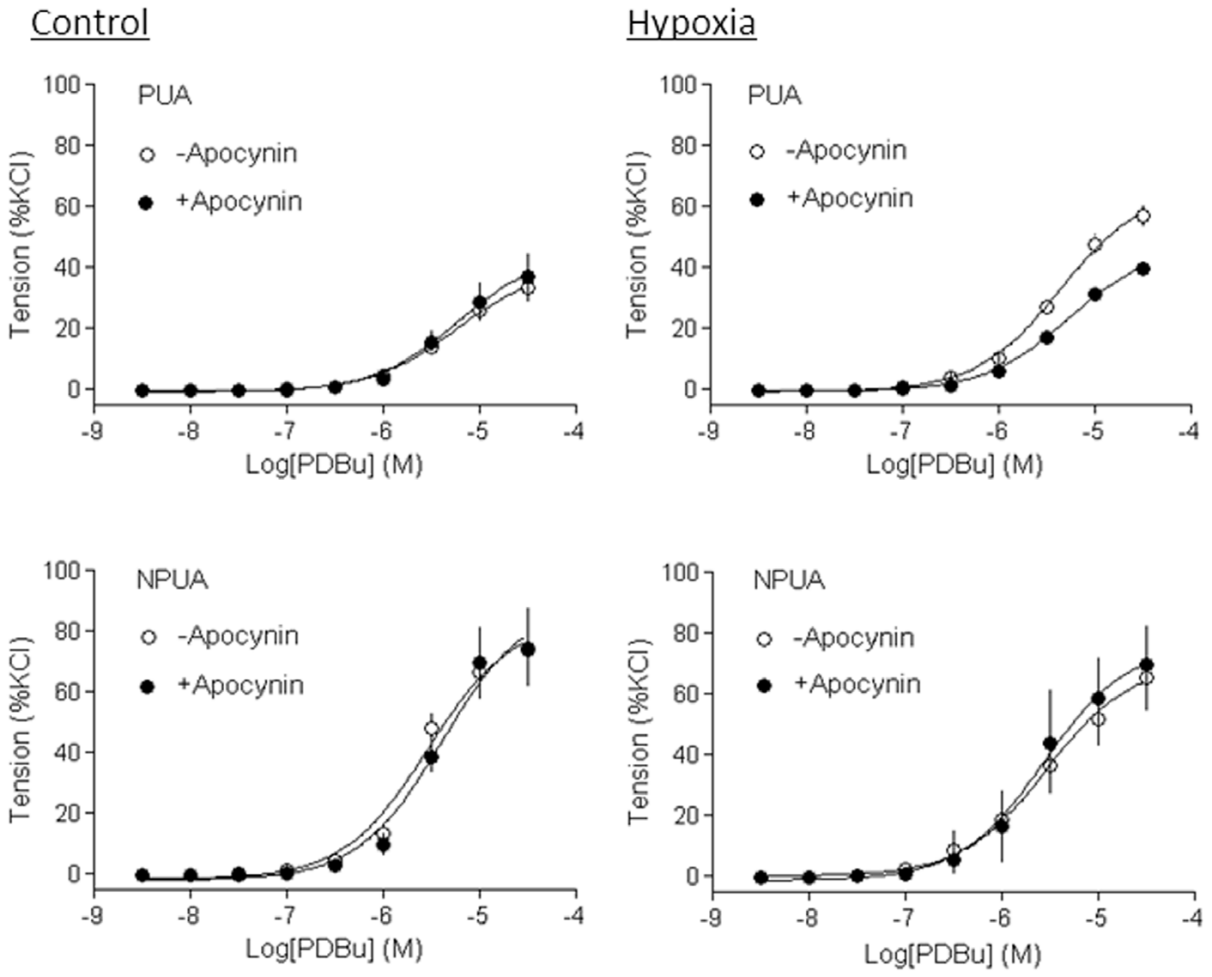
Effect of apocynin on hypoxia-enhanced PKC-mediated contractions of uterine arteries. Uterine arteries were isolated from nonpregnant (NPUA) and near-term pregnant (PUA) sheep maintained at sea level (control) or exposed to high-altitude hypoxia for 110 days. PDBu-induced concentration-dependent myogenic vasoconstrictions were determined in the absence or presence of apocynin (100 µmol/L). Data are means ± SEM of tissues from 4–6 animals.

**Figure 7:**
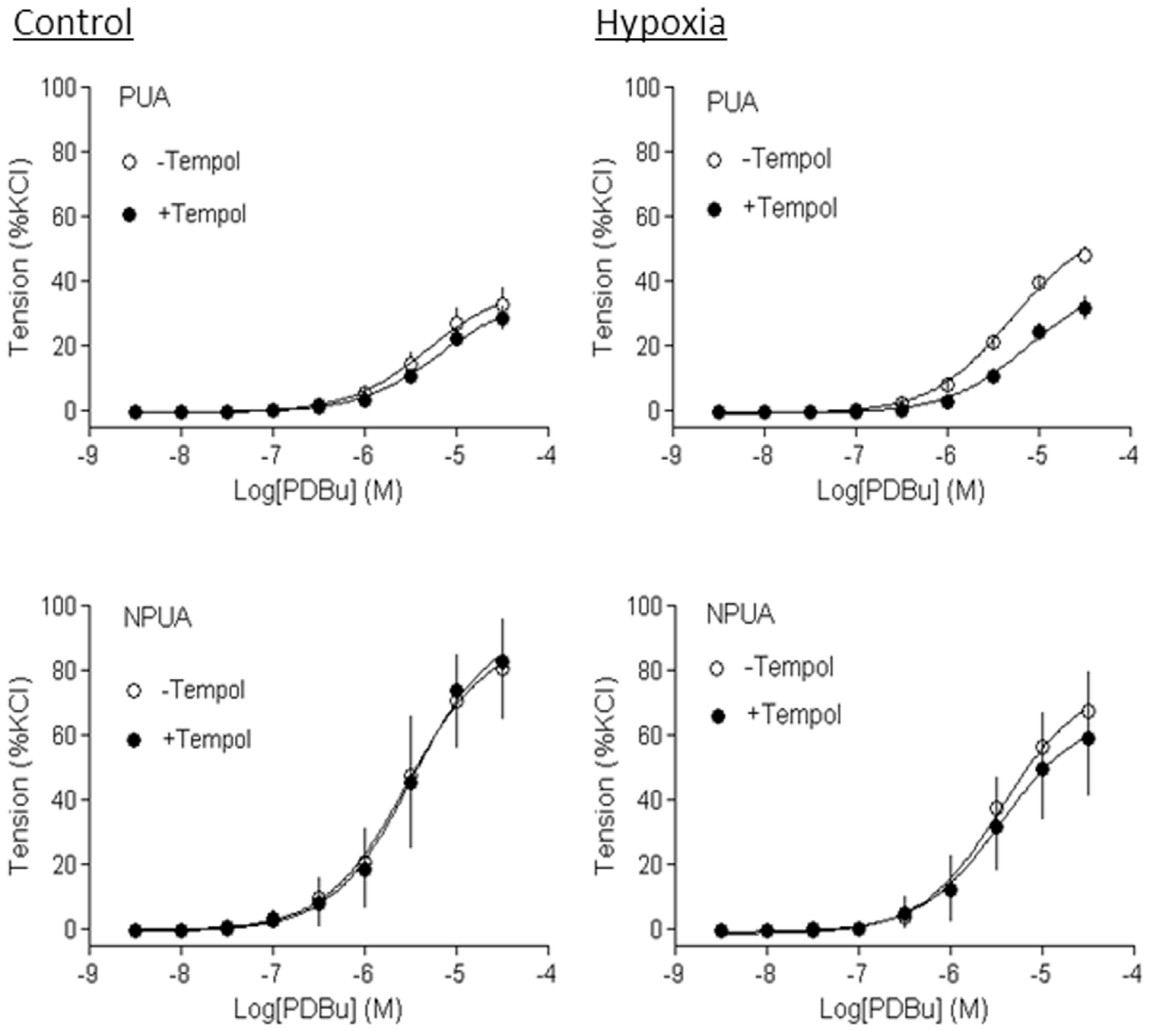
Effect of tempol on hypoxia-enhanced PKC-mediated contractions of uterine arteries. Uterine arteries were isolated from nonpregnant (NPUA) and near-term pregnant (PUA) sheep maintained at sea level (control) or exposed to high-altitude hypoxia for 110 days. PDBu-induced concentration-dependent myogenic vasoconstrictions were determined in the absence or presence of tempol (100 µmol/L). Data are means ± SEM of tissues from 4–6 animals.

Previous studies have demonstrated that PKC plays an important role in pressure-dependent myogenic response of resistance uterine arteries, and chronic hypoxia significantly increased PKC-mediated myogenic reactivity in uterine arteries of pregnant sheep [7], [14], [15], [29]. As shown in Figure 6, PDBu-induced contractions of uterine arteries of pregnant sheep were significantly increased in animals acclimatized to long-term high altitude hypoxia, as compared with the control animals (E_max_: 66.1±2.5% versus 39.7±3.1%, P<0.05). Apocynin had no significant effect on PDBu-induced contractions in normoxic pregnant sheep (E_max_: 45.0±5.6% versus 39.7±3.1%, P>0.05), but significantly decreased the PDBu-induced maximal response in hypoxic animals (E_max_: 46.9±1.5% versus 66.1±2.5%, P<0.05). In the presence of apocynin, there was no significant difference in PDBu-induced contractions of uterine arteries in normoxic and hypoxic pregnant animals (Emax: 45.0±5.6% versus 46.9±1.5%, P>0.05). In nonpregnant animals, neither hypoxia nor apocynin had significant effects on PDBu-induced contractions (Figure 6). Similar results were obtained with a SOD mimetic tempol (Figure 7).

### Inhibition of ROS reversed hypoxia-downregulated BK_Ca_ Channel activity

**Figure 8:**
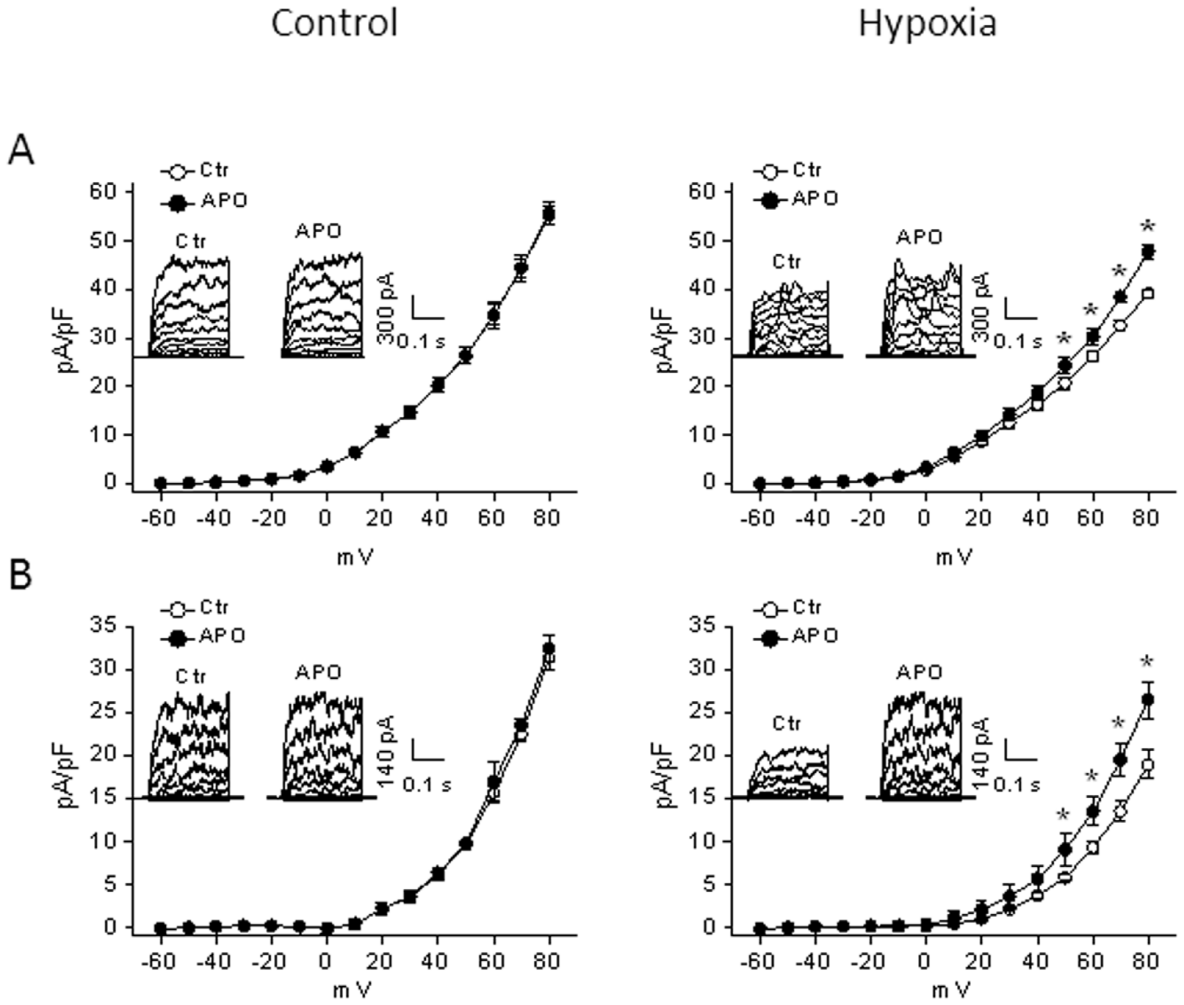
Effect of apocynin on hypoxia-inhibited BK_Ca_ channel activity in uterine arteries. Smooth muscle cells were isolated from uterine arteries of near-term pregnant sheep maintained at sea level (control) or exposed to high-altitude hypoxia for 110 days. **A.** Whole-cell K^+^ currents were recorded in the absence (Ctr) or presence of apocynin (APO, 100 µmol/L). **B.** BK_Ca_ currents in the presence of 4-AP (5 mmol/L) were recorded in the absence (Ctr) or presence of apocynin (APO, 100 µmol/L). Data are means ± SEM of 4–8 cells from 4–6 animals of each group. *, P<0.05, versus -apocynin (Ctr).

Our previous study demonstrated a functional link of the BK_Ca_ channel in pregnancy-mediated downregulation of PKC and myogenic tone in uterine arteries [8]. Figure 8 shows the effect of apocynin on the BK_Ca_ channel activity in uterine arterial myocytes of pregnant sheep. Consistent with the previous finding [14], chronic hypoxia inhibited BK_Ca_ channel activity and significantly decreased whole-cell K^+^ currents in uterine arterial myocytes (55.3±2.0 pA/pF versus 39.2±0.9 pA/pF at +80 mV, P<0.05). Apocynin had no effect on whole-cell K^+^ currents in uterine arteries of normoxic animals (55.6±2.4 pA/pF versus 55.3±2.0 pA/pF at +80 mV, P>0.05), but significantly increased K^+^ currents in uterine arteries of animals acclimatized to long-term high altitude hypoxia (47.1±1.5 pA/pF versus 39.2±0.9 pA/pF at +80 mV, P<0.05). To further determine the specific effect of apocynin on BK_Ca_ channel activity, the voltage-gated K^+^ channel was blocked with 4-aminopyridine. In the presence of 4-aminopyridine, whole-cell K^+^ currents were decreased from 55.3±2.0 pA/pF to 31.2±1.5 pA/pF in normoxic animals, and from 39.2±0.9 pA/pF to 18.9±1.6 pA/pF in hypoxic animals, respectively, and the remaining K^+^ currents represent mainly the component of BK_Ca_ channels. Apocynin showed no effect on the BK_Ca_ channel activity in normoxic animals (31.2±1.5 pA/pF versus 32.3±1.5 pA/pF at +80 mV, P>0.05), but significantly increased it in hypoxic animals (26.5±2.2 pA/pF versus 18.9±1.6 pA/pF at +80 mV, P<0.05). Whereas in the absence of apocynin, there was a significant decrease in the BK_Ca_ channel activity in hypoxic (18.9±1.6 pA/pF), as compared with the normoxic (32.3±1.5 pA/pF) animals (P<0.05), in the presence of apocynin, there was no significant difference in the BK_Ca_ channel activity between hypoxic (26.5±2.2 pA/pF) and normoxic (31.2±1.5 pA/pF) animals (P>0.05). Neither hypoxia nor apocynin affected the BK_Ca_ channel activity in uterine arterial myocytes of nonpregnant animals (data not shown).

## Discussion

The present study presents evidence for the first time that high-altitude chronic hypoxia during gestation enhanced uterine arterial ROS production. Heightened ROS generation coincided with an increase in Nox2 protein expression in uterine arterial walls of pregnant animals. Of importance, the hypoxia-induced impairment of BK_Ca_ channel activity and increase in PKC-mediated myogenic reactivity of uterine arteries in pregnant animals were reversed by the inhibition of ROS production and/or removal of ROS. These findings provide functional evidence of a causative role of heightened oxidative stress in the maladaptation of uterine circulation caused by chronic hypoxia during gestation.

In the present study, our findings that chronic hypoxia increased HIF-1α expression in the uterine arteries, suggest that hypoxia can directly regulate uterine vascular molecular signaling pathways and vascular function. The current findings that chronic hypoxia significantly enhanced ROS production in uterine arteries during pregnancy, are consistent with previous findings in pulmonary arteries exposed to hypoxia [31], [32]. These observations suggest vascular smooth muscle cell can sensor low O_2_ concentration and signal hypoxic HIF-1 by release of ROS, and support the notion that hypoxia causes vascular dysfunction due to an upregulation of ROS levels in the vasculature. ROS consist of a diverse family of small molecules such as superoxide anion (O_2_^−^) and hydrogen peroxide, and the enzyme Nox is the primary generator of ROS in blood vessels. Our present findings that chronic hypoxia significantly increased total Nox activity in pregnant uterine arteries, further suggest that Nox may be the major generator of ROS and contribute to aberrant uterine arterial responses in sheep exposed to chronic hypoxia. Although Nox1, Nox2, and Nox4 were expressed in uterine arteries, chronic hypoxia selectively up-regulated the expression of Nox2 in pregnant animals. This finding suggests that Nox2-derived ROS may functionally attribute to chronic hypoxia-induced alterations of uterine myogenic reactivity and vascular tone. Furthermore, chronic hypoxia enhanced Nox2 protein expression in uterine arterial walls only in pregnant but not in nonpregnant animals, suggesting that sex steroid hormones may participate in regulating Nox2 gene expression. Indeed, Nox expression in human endothelial cells was inhibited by estrogen [33]. Ovariectomy resulted in increased blood pressure and an enhanced oxidative stress in aorta of Dahl salt-sensitive rats due to an increased expression of Nox, which was rescued by estrogen supplementation [34]. In addition, estrogen also regulates Nox activity. It was demonstrated that estrogen attenuated ischemic oxidative damage *via* an estrogen receptor α-mediated inhibition of Nox activation [35]. Therefore, estrogen plays an important role in protecting the cardiovascular system against ROS-mediated adverse impacts. Chronic hypoxia during gestation significantly suppressed the expression of estrogen receptor α in uterine arteries due to heightened promoter methylation [16], [36]. Thus, the inhibition of estrogen on Nox expression and/or activity in uterine arteries is likely to be removed by chronic hypoxia, leading to enhanced ROS generation.

In the present study, we found that chronic hypoxia significantly enhanced Nox2 (gp91^phox^) protein expression in uterine arteries of pregnant animals. It has been shown that Nox2 requires the assembly of at least five additional components for its activation [37]. These additional proteins include the membrane-bound p22^phox^, which helps stabilize the Nox proteins and the cytosolic proteins p47^phox^, p67^phox^, the small GTP_ase_ Rac, and p40^phox^, which together modulate and lead to the activation of the Nox enzyme. Recent studies have demonstrated that acute hypoxia significantly increases Nox activity and translocation of p47^phox^ protein to the plasma membrane in pulmonary arteries. Furthermore, deletion of p47^phox^ gene attenuated hypoxia-induced Nox activation and ROS production in pulmonary arteries [38]. These findings suggest that Nox-associated subunits may play an important role in hypoxia-mediated ROS production. Future studies are needed to further investigate the interaction among these Nox-related proteins and their roles in chronic hypoxia-induced ROS production and increased uterine vascular tone.

Consistent with previous studies [7], [29], the present study demonstrated attenuation of pressure-induced myogenic tone of uterine arteries during pregnancy. The mechanisms of pregnancy-mediated decrease in the uterine myogenic tone are not fully understood. Myogenic tone is an intrinsic property of the smooth muscle and is independent of neural, metabolic, and endothelial influences. Nonetheless, myogenic tone can be modified by different physiological and pathological factors. Our previous study revealed that steroid hormones and upregulated downstream signaling pathways coupled to the hormones during pregnancy played a key role in the attenuated myogenic tone of uterine artery [29]. The present finding that inhibition of ROS generation did not alter pressure-induced myogenic reactivity of uterine arteries in both nonpregnant and pregnant sheep of normoxic animals (data not shown) suggests that basal ROS levels may not contribute to the regulation of uterine vascular tone under physiological conditions. However, elevated levels of ROS resulted in vascular complications in diabetes mellitus and inhibition of ROS production reversed the heightened myogenic tone in diabetic cerebral artery [39], suggesting that increased ROS may contribute to altered vascular myogenic tone under pathophysiological conditions. This notion was supported by our findings that the inhibition of enhance ROS generation resulted in attenuation of pressure-induced myogenic tone of uterine arteries in pregnant animals acclimatized to long-term high altitude hypoxia. The finding that ROS inhibition abrogated the difference in uterine arterial myogenic tone between normoxic and hypoxic pregnant animals suggests a major causative role of increased ROS production in the heightened uterine arterial myogenic tone in response to chronic hypoxia.

Further evidence comes from the finding that the inhibition of ROS production and/or removal of ROS abolished hypoxia-induced up-regulation of PKC-mediated myogenic constriction of uterine arteries in pregnant animals. Previous studies demonstrated that down-regulation of PKC significantly contributed to the reduced myogenic tone of uterine arteries during pregnancy, which was abrogated by chronic hypoxia [7], [15], [16], [29]. The present finding that enhanced uterine myogenic vasoconstriction induced by PKC activation in hypoxic pregnant animals was abolished by a Nox inhibitor apocynin or a SOD mimetic tempol, re-enforces the notion that hypoxia-mediated ROS production is a cause of heightened myogenic reactivity of uterine arteries in pregnant animals.

A possible mechanism of ROS-induced increase in uterine arterial myogenic reactivity is *via* inhibition of the BK_Ca_ channel activity. BK_Ca_ channels play a key role in regulating myogenic tone of uterine arteries in pregnancy, as well as in response to chronic hypoxia [8], [14]. Chronic hypoxia during gestation selectively impaired BK_Ca_ channel activity in uterine arteries of pregnant animals [14]. The present study demonstrated that inhibition of ROS by apocynin reversed the chronic hypoxia-induced suppression of BK_Ca_ channel activity in uterine arteries of pregnant animals, providing a mechanistic link of the BK_Ca_ channel between the elevated oxidative stress and heightened myogenic tone in uterine arteries of pregnant animals acclimatized to long-term high altitude hypoxia. Inhibition of the BK_Ca_ channel activity by ROS has also been observed in recombinant expressing systems, vascular smooth muscle cells and endothelial cells [40]–[44]. ROS likely exert their inhibitory effect on BK_Ca_ channels by targeting a cysteine residue near the Ca^2+^-bowl of the BK_Ca_ channel α subunit to alter Ca^2+^ sensing [43].

### Perspectives

Chronic hypoxia during gestation increases incidence of preeclampsia and fetal intrauterine growth restriction due to maladaptation of the uteroplacental circulation. However, mechanisms underlying these effects remain poorly understood. Heightened oxidative stress is associated with a variety of pathophysiological conditions including preeclampsia. The present study demonstrates that chronic hypoxia during gestation upregulates Nox2 expression in uterine arteries leading to increased ROS production, which in turn results in suppressed BK_Ca_ channel activity and increased uterine arterial myogenic tone. Thus, the present finding provides a mechanistic understanding of heightened oxidative stress in maladaptation of the uteroplacental circulation associated with chronic hypoxia during gestation, and may shed light on a causative factor in pathophysiology of preeclampsia.
